# Supplementary figures and images for: Identification of heat responsive genes in pea stipules and anthers through transcriptional profiling
Source: PLoS One. 2021 Nov 4;16(11):e0251167. doi: 10.1371/journal.pone.0251167 (PMC8568175; doi:10.1371/journal.pone.0251167)

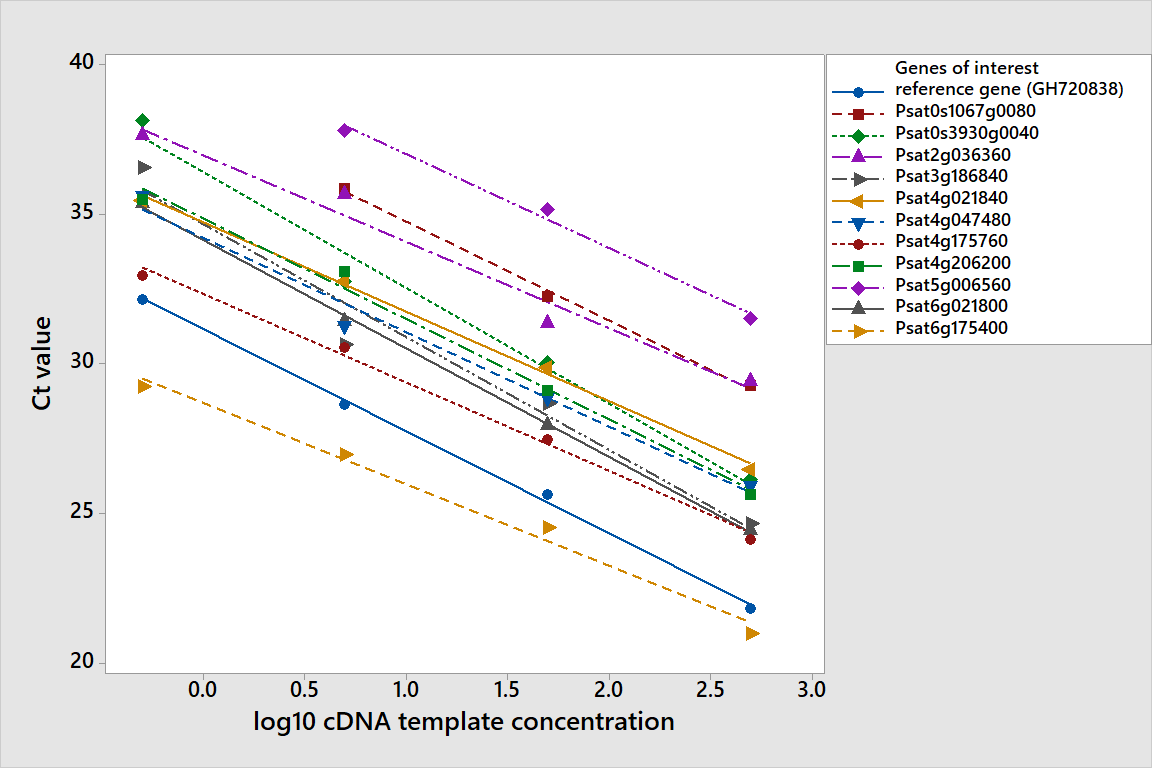

Supplement: S1 Fig — (TIF) [file pone.0251167.s001.tif]
